# Evolution of Flight Muscle Contractility and Energetic Efficiency

**DOI:** 10.3389/fphys.2020.01038

**Published:** 2020-10-09

**Authors:** Tianxin Cao, J.-P. Jin

**Affiliations:** Department of Physiology, Wayne State University School of Medicine, Detroit, MI, United States

**Keywords:** striated muscle, molecular evolution, flight muscle, myofilament proteins, insect, bird, bat, energetic efficiency

## Abstract

The powered flight of animals requires efficient and sustainable contractions of the wing muscles of various flying species. Despite their high degree of phylogenetic divergence, flight muscles in insects and vertebrates are striated muscles with similarly specialized sarcomeric structure and basic mechanisms of contraction and relaxation. Comparative studies examining flight muscles together with other striated muscles can provide valuable insights into the fundamental mechanisms of muscle contraction and energetic efficiency. Here, we conducted a literature review and data mining to investigate the independent emergence and evolution of flight muscles in insects, birds, and bats, and the likely molecular basis of their contractile features and energetic efficiency. Bird and bat flight muscles have different metabolic rates that reflect differences in energetic efficiencies while having similar contractile machinery that is under the selection of similar natural environments. The significantly lower efficiency of insect flight muscles along with minimized energy expenditure in Ca^2+^ handling is discussed as a potential mechanism to increase the efficiency of mammalian striated muscles. A better understanding of the molecular evolution of myofilament proteins in the context of physiological functions of invertebrate and vertebrate flight muscles can help explore novel approaches to enhance the performance and efficiency of skeletal and cardiac muscles for the improvement of human health.

## Introduction

Flying is an energetically demanding activity for animals. Muscle-powered flights have evolved in the classes of *Insecta* (insects) and *Aves* (birds) and in the *Chiroptera* order of mammals (bats). It is interesting to investigate how flight muscles of these phylogenetically distant species have evolved to meet the requirement of highly efficient and sustainable contractions to power their wings during flight. Insect and vertebrate flight muscles are both striated muscles with similar sarcomeric structure, and myofilament motor and regulatory proteins. To compare their similarities as well as unique features together with non-flight muscles for convergent evolutionary selections can help provide insight to the molecular basis of the contractility and energetic efficiency required for flight muscles.

## Evolution of Powered Flight in Insects, Birds, and Bats

The flight capacity of insects, birds, and bats is a typical example of convergent evolution. These animals have independently evolved from different ancestors at different times with analogous functionality of powered flight as results from similar natural selective pressures (Gleiss et al., 2011).

### Origins of Flight

**FIGURE 1:**
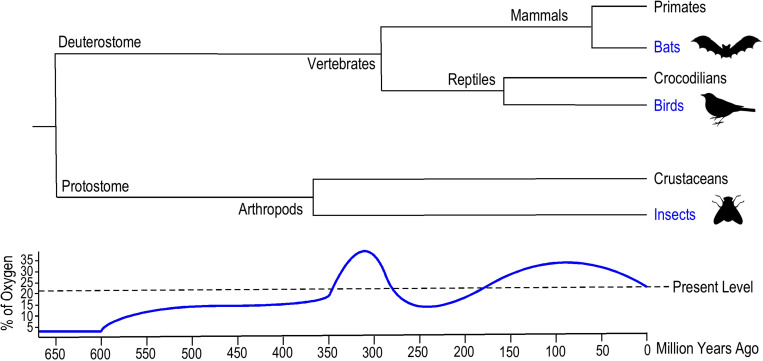
Evolutionary emergence of insects, birds, and bats. The phylogenetic tree summarized from literature data (Nelson and Tidwell, 1987; Tokita et al., 2012; Brusatte et al., 2015) illustrates the geological times when the *Insecta* and *Aves* classes and *Chiroptera* order of flying animals emerged. Insects evolved flight around 400 million years ago (Mya), ancient flying birds appeared 150 Mya and bats emerged around 50 Mya. The historical atmospheric oxygen content curve (Dudley, 1998) illustrates the similar hyperoxia environments when analogous aerial locomotion emerged in these flying animals.

Certain common environmental factors might have been critical to the emergence and convergent evolution of animal flight. Geophysical data indicate that dramatically elevated atmospheric oxygen level occurred twice in the history of Earth (Lyons et al., 2014). The first rise of oxygen levels to 35% was ∼360 million years ago (Mya) from plant terrestrialization and global carbon deposition during late Devonian to late Carboniferous eras when flying insects emerged (Dudley, 2000), and the second rise of oxygen level to 25–30% was in the late Jurassic and Cretaceous eras when flying vertebrates, i.e., birds (∼150 Mya) and bats (∼52 Mya) emerged (Figure 1) (Dudley, 2000).

Wingless insects emerged 395–390 Mya, and although the origin of flying insects is unclear, the ancestor was likely a pterygote hexapod (winged insect) as indicated by the earliest fossils dated from ∼325 Mya (Figure 1) (Nelson and Tidwell, 1987). The morphological origin of wings remains debatable since there is no direct evidence of transitional forms between wingless and winged insects. A compelling theory based on the primitive fossil record of ancestral pterygote insect nymphs proposes that the origin of insect wings emerged from a combination of the dorsal part of the thorax and the body wall (Prokop et al., 2017). The extended thoracic lobes developed articulation to enable gliding, and musculature formed later to power the flapping of wings.

With the increased metabolic demand of flapping wings during flight, the thoracic muscles of insects have the highest mass-specific rate of oxygen consumption in comparison to other tissue types (Harrison and Lighton, 1998). Higher partial pressure of oxygen (PO_2_) compensated for the diffusional limits in insect tracheal systems during respiration, reducing constraints on maximum body size, and the atmospheric hyperoxia may have played a role in the evolution of gigantism (Dudley, 1998). All giant arthropods from the late Paleozoic era went extinct by the end of Permian, possibly due to the decline of atmospheric oxygen concentration (Dudley, 1998).

Flying birds emerged later than flying insects (Figure 1). Fossil evidence indicates that birds evolved from small carnivorous dinosaurs during the late Jurassic era ∼150 Mya (Brusatte et al., 2015). *Archaeopteryx* is thought to be a transitional form between non-avian feathered dinosaurs and modern birds (Xu et al., 2011). Notably, the second rise of atmospheric oxygen level occurred during the emergence of avian flight (Dudley, 2000). There are two hypotheses for the evolutionary origins of avian flight: the “direct aerial descent” hypothesis and the “fundamental wing-stroke” hypothesis (Askew and Ellerby, 2007). The primary debate between these two hypotheses is whether avian flight evolved from gliding or wing flapping ancestors. Nonetheless, the higher atmospheric PO_2_ presumably supported the evolution of aerodynamic force production and enhanced oxygen perfusion for avian pectoral muscles to power wing flapping flight.

Bats are the only extant mammalian lineage with the ability of powered flight. The emergence of bats can be traced to the early Paleocene to late Cretaceous eras ∼52 Mya (Figure 1), which was under a hyperoxic environment similar to when avian flight emerged. Molecular phylogenetic analysis demonstrated a monophyly of bats, suggesting the origin of powered flight in bats was from a single ancestral source (Tokita et al., 2012). It is widely accepted that flying mammals evolved from small arboreal mammals rather than terrestrial runners, and the microchiropteran fossil of *Onychonycteris* from 52 Mya during the Eocene period supports this perspective (Bishop, 2008). *Onychonycteris* had longer hind legs and shorter forearms compared to modern bats, implicating primitive short flights from tree to tree. This “tree-down” theory of the evolution of flight proposes that bats gained flying ability through dropping down from a tree instead of from fast running for a ground take-off (Bishop, 2008). No intermediate fossil evidence directly links bats to any flightless ancestors, suggesting a rather rapid emergence of flying bats. The evolution of the unique musculature within the wings of bats (Cheney et al., 2017) remains to be investigated.

Proposed as an essential environmental factor to promote the evolution of animal flight, the high atmospheric PO_2_ might have increased the systemic metabolic potential and physical activities of animals to reach the requirement of flight. Besides the atmospheric hyperoxia effect on the emergence of flight, the higher air density in those geological eras might have also favored the aerodynamic lifting effects of flapping and airfoil of wings (Combes and Daniel, 2003). In addition to increasing muscle mass for higher power output during flight, avian bones were fused to eliminate joints and reduced in mass (Foth et al., 2014). These adaptations of flying species are centered around increasing muscle power and energy supply as well as work efficiency to sustain the high cost activity of flight.

### Independent Evolution of Convergent Traits in the Three Flying Animal Lineages

The capability of flight enables animals to reach additional food sources, to pass through physical barriers for living in more favorable habitats, and to escape from ground-living predators. Although many species of animals have evolved gliding ability, the only extant groups that acquired powered flight are insects, birds, and bats. The evolution of animal flight is one of the fascinating examples of phenotypic convergence, in which distant lineages have evolved the same physiological function to adapt to similar environmental selections.

Insects were the first group of animals that evolved wings and the ability to fly with fossil records suggest that they only evolved once (Ross, 2017). A generally accepted hypothesis proposes that insect flight originated on land rather than on the surface of the sea. Fossils of the most primitive insect-like springtail *Rhyniella praecursor* from 407 Mya support this hypothesis (Dunlop and Garwood, 2018). Springtail, as a sister group of insects, is a wingless six-legged hexapod. A unique structure of springtails, the furcula underneath their body, is able to propel the animal into the air to avoid hazardous environments. The airborne ability of the ancient springtails indicated the possibility of winged insects to evolve on land (Engel and Grimaldi, 2004). The wing structure of insects evolved from extending thoracic lobes which were used for thermoregulation in ancestral pterygotes. Subsequently, the extended thoracic lobes were articulated for the gliding function, and muscles emerged to power flapping for flight (Ross, 2017).

Vertebrates evolved flight later in separate lineages. Birds are one of the most divergent classes of modern vertebrates, due to their ability to fly. Fossil evidence and molecular evolutionary studies in recent decades demonstrate that the origin of birds was from theropod dinosaurs during the Jurassic period (Brusatte et al., 2015). The small, light-weighted, feathered, and winged body plan first emerged in the Mesozoic period. It is hypothesized that the origin of powered avian flight emerged within the surviving species of theropod ancestors after the mass extinction (Brusatte et al., 2015). Along with an increase in the mass of pectoral muscles, postcranial skeleton pneumatization (hollow air-filled bones) and bone fusions emerged in birds to lighten the body weight during the evolution of flight (Britt et al., 1998).

Bats are the only flying mammal, evolved from a gliding ancestor who climbed trees and launched themselves into the air to extend flight distance (Bishop, 2008). Fossil records show the emergence of bats having full ability of flight ∼52 Mya. Bats have evolved into two suborders: *Microchiroptera* and *Megachiroptera*. These two groups have significant differences in size, diet, and habitat. *Megachiroptera* (megabats) have much larger body sizes compared to *Microchiroptera* (microbats). Microbats evolved the ability of echolocation using high frequency sounds to detect small preys such as insects, while megabats are nectarivores (Dechmann et al., 2006).

The convergent evolution of flight in insects, birds, and bats share the common adaptation of muscle-powered wing movements to generate the sustainable aerodynamic lift that is energetically demanding. Therefore, the evolution of the flight muscles in these phylogenetically divergent species should reflect adaptations due to the selection for high efficiency power generators.

## Anatomic and Physiologic Features of Insect, Bird, and Bat Flight Muscles

Insect muscle and vertebrate striated muscles are analogous organs, whereas avian and bat flight muscles are homologous organs specifically selected for flight. Therefore, comparison among muscles of the three independently evolved groups of flying animals can provide insights into how natural selection drives the evolution of analogous structures and their functions as well as radical divergences.

### Flight Muscles of Insects

**FIGURE 2:**
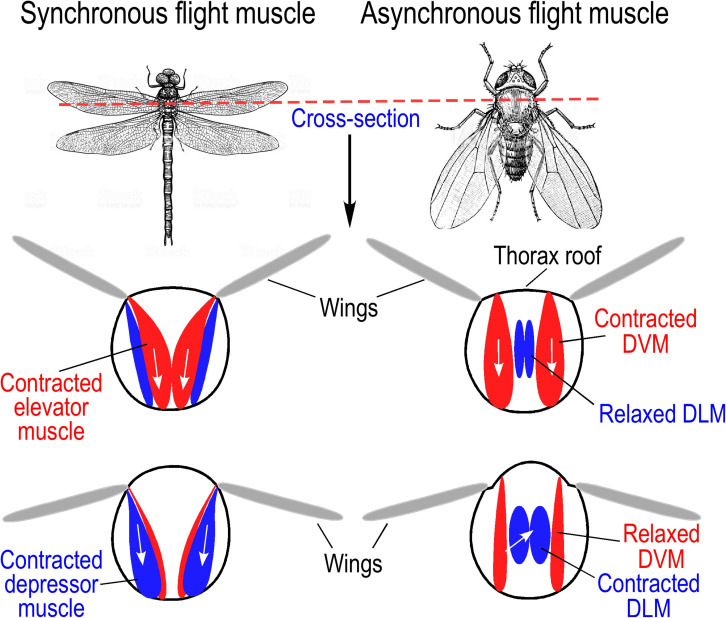
Direct and indirect insect flight muscles. **(Left)** Wing movement driven by synchronous direct flight muscles. Larger insects, such as dragonflies and locusts, use direct flight muscle for wing beating during flight. Contraction of elevator muscles pulls wing up, and depressor muscles pull wings down. **(Right)** Wing movement of fruit fly driven by asynchronous indirect flight muscles. Dorsal vertical muscles (DVM) pull on the thorax roof to produce upstroke of wings while stretching the dorsal longitudinal muscles (DLM). Subsequent contraction of DLM causes shortened anterior and posterior ends of the thorax resulting in downstroke of wings and DVM stretching to induce the next stretch-activated cycle. The illustrations were summarized from literature information (Iwamoto, 2011).

Insects are the only flying invertebrates on Earth, and they evolved the ability of flight with small body sizes to maximize the frequency of wings beating (Conley and Lindstedt, 2002). Insect flight muscles are located in the thorax and function in a direct or indirect manner (Bullard and Pastore, 2011; Baumler et al., 2018) (Figure 2) with synchronous or asynchronous contractions (Ellington, 1985).

Synchronous direct flight muscles are found in lower species of insects like locusts (*Orthoptera*) and dragonfly (*Odonata*), which have wing beats at lower frequencies (<100 Hz). Similar to the actions of avian pectoral muscles, contraction of elevator muscles in the thorax lift the wings, and depressor muscle contractions power the wings (Baumler et al., 2018) (Figure 2). The mode of contraction and relaxation of synchronous insect flight muscles is similar to that of vertebrate skeletal muscle, in which contraction is induced by depolarization of myocyte membrane in response to impulses arriving from motor nerves, and the contraction-relaxation cycle depends on the rise and decay of cytosolic [Ca^2+^] (Ellington, 1985; Iwamoto, 2011).

Most of the extant species of insects have asynchronous indirect flight muscles (IFMs) to power high frequency wing beating via deformation of the thoracic exoskeleton rather than directly driving the movements of wings (Conley and Lindstedt, 2002). With conserved striated muscle structure, asynchronous IFMs have evolved with a mechanism of stretch activation. There are two sets of IFMs in the thorax, the dorsal longitudinal muscle (DLM) and the dorsal ventral muscle (DVM). The two sets of muscles are arranged in cross orientations and work together to drive oscillated thoracic distortions and wing beats (Conley and Lindstedt, 2002). When taking off, a nerve impulse triggers the DVM to contract, which deforms the thorax, causing a stretch of DLM to induce a delayed stretch activation. The delayed contraction of the DLM in turn causes the DVM to extend and is stretch activated (Deora et al., 2017) (Figure 2). This cycle of stretching and contracting is repeated at high frequency with low frequency stimulating impulses from motor nerves, by which an intermediate rise in cytosolic [Ca^2+^] will sustain continuous oscillations of contraction and relaxation of insect asynchronous IFM (Iwamoto, 2011).

### Avian Pectoral Muscles

Bird flight is primarily powered by the pectoralis muscles that move the humerus bone of the wing around the shoulder. The pectoralis muscles of most adult birds take up approximately 8–11% of the total body mass (George and Berger, 1966; Biewener, 2011). The primary role of these large skeletal muscles is to produce mechanical force for downstrokes of the wing during flight (Poore et al., 1997). Another important flight muscle of birds is the supracoracoideus that is about one-fifth of the mass of the pectoralis. The smaller supracoracoideus muscle is primarily responsible for elevating the wings during upstrokes, particularly important for slow and moderate speed movements as well as for hovering at fast wing movements (Crandell and Tobalske, 2015).

To produce sufficient aerodynamic power to sustain flight, avian flight muscles need to continuously contract at high frequencies with substantial work and with high energy costs. The fiber-type compositions of avian flight muscles vary in different species. The pectoralis contains mainly fast-oxidative fibers (∼85% in pigeons) and a small portion of fast-glycolytic fibers, while in supracoracoideus, the proportions of fast-glycolytic fibers and fast-oxidative fibers can be very different among species (Biewener, 2011).

### Bat Flight Muscles

Like in most flying birds, bats have a keel on the sternum where the large pectoral muscles attach (Gaudioso et al., 2017). Gross anatomy and electromyography studies have also identified numerous muscles in the arm and forearm of bats with roles in powering wing movement and controlling wing shape and orientation during flight (Hermanson and Altenbach, 1981). Similar to that of birds, the pectoralis muscle of a bat is the major muscle to drive the downstroke of wings during flight, and the biceps brachii muscle acts in folding the wings at the elbow during wing downstrokes (Von Busse et al., 2012). Bats have evolved wings for flight from their forelimbs with joints within the digits. Those joints affect three-dimensional wing shape to control aerodynamic force generation (Hedenstrom and Johansson, 2015). Since a large number of muscles are involved in controlling aerodynamics during bat flight, the energy cost associated with the pectoralis muscle is a smaller portion of the total of flight muscles than that of birds (Cheney et al., 2014).

Unlike the flapping wings of birds and insects, bats have evolved unique wing structures that are more like the patagia of gliding animals. Bat wings are formed with membranous skin called plagiopatagium covering the bones of forelimb and connecting the areas between the limb, digits and even the tail. The majority of the surface area of bat wings is skin that varies in thickness between 27 and 270 μm depending on the species. The skin of a bat wing is complex and unique, and is composed of bundles of elastin and embedded tiny skeletal muscles named plagiopatagiales (Skulborstad et al., 2015). The elastin bundles are oriented in a predominantly spanwise direction, supporting a role in facilitating folding and unfolding of the wings during flight (Cheney et al., 2017). Studies of 130 bat species found five different muscle arrays in the plagiopatagium of wings that potentially control the membrane stiffness (Cheney et al., 2017). With the thin and compliant nature of skin, the muscle arrays and elastin bundles control the shape and fine-tune the tension of the membranous wings to adjust aerodynamics during flight.

## Contractility and Regulatory Mechanisms of Different Flight Muscles

Flight muscles of insects, birds, and bats evolved independently with functional convergence. Many of their anatomic and physiologic similarities reflect the adaptation to common selective pressures such as high power output with energetic efficiency for sustainable flights. On the other hand, the unique properties of insect, avian, and bat flight muscles such as resting sarcomere length, passive stiffness, and the mode of Ca^2+^-activated or stretch-activated contractions indicate adaptations to specific selective pressures for a particular type of flight activity (Cutts, 1986; Cao et al., 2019).

### Common Features of Invertebrate and Vertebrate Striated Muscles

**FIGURE 3:**
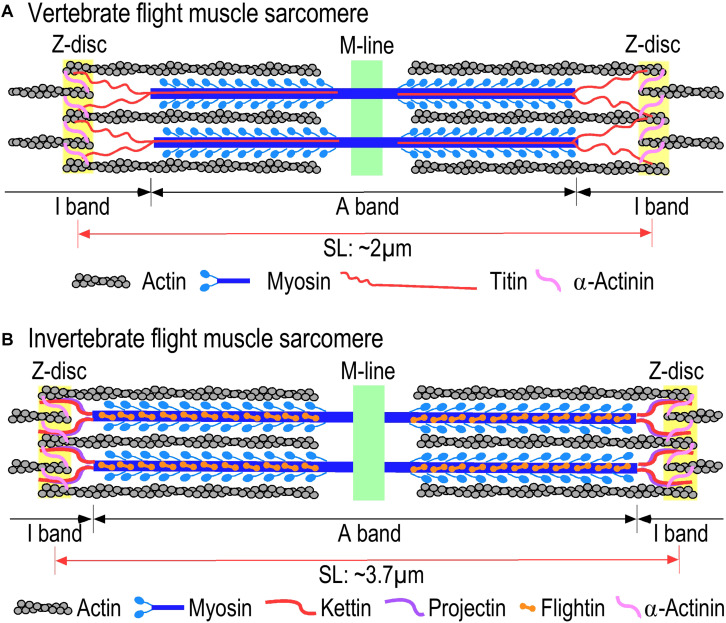
Comparison of sarcomere structures of **(A)** vertebrate and **(B)** invertebrate flight muscles. Invertebrate and vertebrate flight muscles contain similar sarcomeric structures consisting of overlapping myosin thick filaments and actin thin filaments with homologous but diverged scaffolding proteins. Invertebrate indirect flight muscles (IFM) have longer sarcomeres compared to vertebrate flight muscle and narrower I-bands in comparison with the vertebrate sarcomeres. The illustrations were summarized from literature information (Hooper et al., 2008; Lange et al., 2020).

While insects, birds, and bats evolved independently from different ancestral lineages, their flight muscles are analogous striated muscles (Iwamoto et al., 2002; Homberger and de Silva, 2015; Cheney et al., 2017). Vertebrate skeletal muscles and insect IFM have been extensively studied as representatives of striated muscles that contain bundles of myofibrils consisting of tandem repeats of sarcomeres as the contractile unit (Iwamoto, 2011; Sweeney and Hammers, 2018). Reflecting similar mechanisms of function, the structural organization of the sarcomere is similar in flight muscles of insects, birds, and bats with overlapping myosin thick filaments and actin thin filaments and the associated scaffolding and regulatory proteins (Figure 3) (Cutts, 1986; Cao et al., 2019). In a sarcomere, myosin thick filaments are anchored at the M-line, actin thin filaments are anchored at the Z-disk and titin or titin-like elastic proteins linking the thick and thin filaments to constitute a structural continuity (Figure 3). Based on the nature of striated muscles as a highly differentiated tissue type, the contractile machinery and regulatory mechanisms for the contraction and relaxation of insect, bird, and bat flight muscles are largely conserved from their common ancestor that lived 500–700 Mya (Cao et al., 2019).

The myofilament protein contents in both invertebrate and vertebrate flight muscles are conserved, similar to that in other striated muscles (Figure 3). They all contain F-actin-activated myosin motors and the thin filament-associated regulatory proteins tropomyosin and troponin (Tn). The Tn complex is composed of three protein subunits, the Ca^2+^ binding subunit troponin C (TnC), the inhibitory subunit troponin I (TnI), and the thin-filament anchoring subunit troponin T (TnT) (Gordon et al., 2000; Jin et al., 2008). When the muscle contracts, depolarization of plasma membrane of myocytes induces a small amount of Ca^2+^ influx and triggers the release of a more substantial amount of Ca^2+^ from the sarcoplasmic reticulum (SR). The increase in cytosolic [Ca^2+^] promotes the binding of Ca^2+^ to TnC and induces a series of conformational changes in Tn and tropomyosin, allowing the myosin heads to form strong cross bridges with the actin thin filament to activate myosin ATPase and generate power strokes. Muscle relaxation occurs when free Ca^2+^ decreases by the work of SR and plasma membrane Ca^2+^ pumps that remove Ca^2+^ from the cytosol resulting in Ca^2+^ dissociation from TnC (Gordon et al., 2000; Iwamoto, 2011).

Based on the conserved subcellular structures, biochemical basis and functions, unique features of the contractile and regulatory mechanisms of insect, avian, and bat flight muscles can provide insights into the evolution of their contractility and energetic efficiency.

### Synchronous Flight Muscle of Insects

The fact that the flight muscles of lower insects such as locusts and dragonflies with wing beating frequency slower than 100 Hz have synchronous flight muscles that function like the non-flight muscles of insects and vertebrate striated muscles (Snelling et al., 2012) suggests that synchronous direct flight muscle was originally present and asynchronous IFMs may have evolved from them to sustain higher frequency wing movements and potentially with higher energetic efficiency.

The contraction and relaxation of synchronous flight muscles of insects are via the same mechanism as that of vertebrate skeletal muscle involving synchronous excitation-contraction coupling (Peron et al., 2009) as outlined earlier. Contraction is initiated by motor neuron-stimulated excitations at neuromuscular junctions leading to depolarization of the myocyte plasma membrane and a Ca^2 +^ influx that induces the release of larger amounts of Ca^2+^ from the SR. The rising free Ca^2+^ binds to TnC, which induces conformational changes in the Tn complex and tropomyosin in the actin thin filaments, leading to activation of myosin ATPase. ATP hydrolysis generates a power stroke of the myosin head to shorten the sarcomere and contract the muscle. During relaxation, the cytosolic Ca^2+^ is pumped back to SR and extracellular space. With this mechanism, the rate of Ca^2+^ cycling is limited by the rates of Ca^2+^ diffusion and SR Ca^2+^ pumps, restricting the wing beat frequency of insects with synchronous flight muscles to <100 Hz (Iwamoto, 2011). Consistent with the conserved mechanism of contraction and relaxation, the resting sarcomere length of the synchronous flight muscles of locusts is ∼3.5 μm, similar to that of vertebrate skeletal muscles (Nave and Weber, 1990).

### Insect Asynchronous IFM and Stretch Activation

Representing a more advanced adaptation to flight, asynchronous IFM of small insects has evolved with a unique stretch activation for high frequency contractions. To generate high frequency wing beats, low-frequency neuronal impulses activate asynchronous IFM to produce an increase in cytosolic Ca^2+^ to an intermediate level that does not fluctuate during subsequent contraction and relaxation. Instead of the Ca^2+^ wave-dependent myofilament activation–deactivation cycles, the contraction and relaxation of insect asynchronous IFMs utilize the mechanism of mutual stretch activations of two pairs of anatomically cross-oriented thoracic muscles, of which the contraction of one set stretches the other set to activate subsequent contraction and the cycle repeats leading to autonomous oscillations (Squire, 1992) (Figure 2). This mechanism avoids the rate limitations from the equilibrium dynamics of Ca^2+^ transient and pump-driven Ca^2+^ cycling, allowing faster wing beating without high cost work in Ca^2+^ handling (Dickinson et al., 2005).

Insect IFMs are also striated muscles similar to vertebrate skeletal muscle with sarcomeric structure and conserved myofilament proteins, myosin, actin, troponin and elastic scaffolding proteins (Iwamoto et al., 2002) (Figure 3). The resting sarcomere length of insect IFMs ranges from 3.7 μm in *Drosophila* (Bullard et al., 2002) to 2.7–2.9 μm in bee (Nave and Weber, 1990). Different from vertebrate striated muscles, high passive stiffness is a characteristic feature of insect asynchronous IFMs together with narrow I-bands that do not show significant change in length during contraction and relaxation (Hao et al., 2006; Henderson et al., 2017). The apparently rigid sarcomeric structure is likely an adaption for the myofilaments to sense the stretch signal during stretch activations.

The sarcomeric myofilament motor functions in vertebrate and insect muscles both require myosin in the thick filament interacts with actin in the thin filament under the Ca^2+^-dependent regulation of Tn (Gordon et al., 2000; Sweeney and Hammers, 2018; Cao et al., 2019). Increasing evidence suggests that conformational distortions of both myosin in the thick filament and regulatory proteins in the thin filament play roles in the stretch activation of asynchronous IFMs (Perz-Edwards et al., 2011; Iwamoto and Yagi, 2013). Studies in *Drosophila* IFM demonstrated that the myosin converter domain is critical to the generation of sufficient aerodynamic power for flight (Swank et al., 2002). Disruption of myosin converter domain decreases stretch activated tension primarily via decreasing the passive stiffness of the muscle fibers (Wang et al., 2014). The *N*-terminal domain of myosin regulatory light chain plays a structural role in determining the number of strongly bound cross-bridges of myosin and power generation (Miller et al., 2011). A study in *Drosophila* showed that the alternatively spliced *N*-terminal region of myosin heavy chain is a regulatory site for optimizing power generation in IFM (Swank et al., 2004). Real-time X-ray diffraction serial imaging studies revealed Tn bridges that mechanically tug tropomyosin aside as the fiber from *Lethocerus* IMF is stretched (Perz-Edwards et al., 2011). Different alternative splice forms of TnT are found to alter the Ca^2+^ sensitivity and power output of insect IFM (Marden et al., 1999). While these molecular mechanisms may jointly contribute to the stretch activation of insect IFMs, a hypothesis worth testing is that the tension signal of mechanical stretch of sarcomeric myofilaments may alter thin filament conformation and increase troponin’s sensitivity to the constant intermediate level of cytosolic Ca^2+^, which activates actomyosin ATPase and cross bridge cycling.

### Avian Flight Muscles

Compared to other flying species, flying birds have massive pectoralis and supracoracoideus muscles to power the downstroke and upstroke of wings during flight. Avian pectoral muscles contain predominantly fast twitch fibers and have been studied as a classic vertebrate skeletal muscle for their typical myofibril and sarcomeric structures and myofilament protein contents (Cutts, 1986). The determining mechanism(s) evolved uniquely in the avian pectoral muscle in adaptation to sustained flight such as that during long distance non-stop migration remains to be established. Hovering is a unique mode of flight where the birds maintain nearly stationary positions in the air (Vejdani et al., 2018). Hummingbirds, the smallest avian species, are the only birds that can sustain hovering. Their small body size and proportionally larger pectoral muscles allow them to sustain aloft and hovering (Achache et al., 2017). In comparison with other birds, hummingbirds have significantly higher frequency wing beats (∼34 Hz) with much lower force and strain generated by the pectoralis muscles (Tobalske, 2016). The duration of a neural impulse during hummingbird pectoral muscle activation is shorter than that of other birds, corresponding to a shorter time for excitation-contraction coupling during high frequency wing beats and presumably less Ca^2+^ influx into myocytes with lower activation of myosin motors (Tobalske, 2016).

Avian flight muscles have typical sarcomere structures (Figure 3B). The resting sarcomere length is 2.08–2.14 μm in chicken pectoralis (McGinnis et al., 1989; Burkholder and Lieber, 2001), ∼1.89 μm in hummingbird pectoralis muscle, and ∼2.5 μm in hummingbird leg muscle. In contrast, sarcomeres of mammalian skeletal muscles are longer, ∼2.3 μm in rat slow and fast skeletal muscles, 2.27 μm in rabbit, and 2.64 μm in human (Burkholder and Lieber, 2001; Reiser et al., 2013). The longer resting sarcomere would be closer to the optimal length for maximum force production (Reiser et al., 2013). Therefore, the functional significance for the trade-off of shorter sarcomere length in avian pectoral muscles remains to be investigated. Despite the different contractile demands of flight muscles between species, the myosin heavy chain (MHC) isoform is remarkably uniform in pectoral muscles of most avian species with very limited variation (Velten and WelchJr., 2014; Tobalske, 2016). Since the same myosin motor is able to meet the general requirement of various types of powered flight, the different contractile and performance features of the flight muscles of different avian species may depend on adaptations and variations in the thin filament regulatory proteins.

The *N*-terminal region of TnT is variable in structure due to genetic variations, alternative splicing, and proteolytic modification, which produces long range conformational modulations to modify the function of Tn and muscle contractility (Wang and Jin, 1998; Wei and Jin, 2016). The avian fast TnT gene has evolved with up to seven unique P exons encoding repeating motifs of His-Glu rich sequences in the *N*-terminal variable region (Jin and Samanez, 2001). In addition to the alternative splicing to exclude embryonic exons to reduce the size and negative charge of the *N*-terminal region seen in the developmental regulations of both skeletal muscle and cardiac TnT isoform genes across vertebrate species (Wang and Jin, 1997; Jin et al., 2008), avian pectoral muscle fast TnT undergoes a parallel post-hatch alternative splice-in the P exons to increase the size and negative charge of the *N*-terminal variable region (Ogut et al., 1999). This phenotype is not seen in the leg muscles and is concurrent with a rapid growth of pectoral muscle mass (Jin and Samanez, 2001). The developmental and functional significances of this intriguing flight muscle-specific adaptation remain to be established.

### Pectoral and Wing Muscles of Bats

Bat flight muscles have conserved structure and contractile and regulatory mechanisms similar to that of other mammalian striated muscles. The resting sarcomere length of bat pectoral muscle is ∼2.2 μm and is similar to that of rat skeletal muscles (Mathieu-Costello et al., 1992). A feature of bat flight is that the recruitment of pectoralis muscle fibers is modulated by flight speed similar to that of birds (Konow et al., 2017). Power production is associated with changes in flight speed (Hermanson et al., 1998). Varied flight speeds of different bat species might be due to diverse fiber compositions. Pectoralis muscle of insect-feeding bats contains only non-fatigable fast oxidative-glycolytic fibers, whereas fruit-feeding bat pectoralis muscle contains multiple types of fiber (Hermanson et al., 1998; Konow et al., 2017).

Bat wings have the ability to adjust their morphology in different aerodynamic conditions using the plagiopatagiales muscles. These muscles originate from the skeleton and are inserted in the plagiopatagium membrane. There are also intramembrane muscles arranged in parallel to the chord, which are not connected to bone. Contractions of these muscles generate tension in the plagiopatagium membrane and trailing edges synchronously to shape bat wings for resisting aerodynamic load during wing beat cycles for a steady flight (Cheney et al., 2014).

The apparent lack of flight-specific adaptations in bat pectoral muscles may be a reason that bats need to exhaustively utilize muscle power and whole body metabolism to sustain flight (O’Shea et al., 2014). This mechanism is different from the evolutions of insect and avian flight and puts bats consistently under metabolic stress that generates a unique immunological state for serving as reservoir hosts of various viruses that are pathogens of human and other mammals (O’Shea et al., 2014). This notion implicates that the evolution and adaptation of flight muscles may have systemic impact on the life of animals, with which intensive exercise and utilization of skeletal muscles provides a potential mechanism to produce immunotolerance (Calder and Yaqoob, 1999) for the treatment of inflammatory disease in humans.

## Energetic Efficiency of Insect, Bird, and Bat Flight Muscles

Muscle-powered locomotion is a major energy expense of animal life, and various strategies have evolved to reduce the cost of muscle contraction. Besides increasing the mass of flight muscles and improving wing structure and reducing body weight to reduce muscle work, energetic efficiency of flight muscle contraction and relaxation is essential for the flying species to sustain the high cost function of powered flight (Ellington, 1985; Ward et al., 2001; Hedenstrom and Johansson, 2015). Muscle energetic efficiency can be described as the ratio of mechanical power to energy expenditure, which shows the proportion of chemical energy that is converted into mechanical work (Morris et al., 2010). The independently evolved flight muscles of insects, birds and bats have acquired different features in contractile kinetics and ATP supply for a balanced energy expenditure.

### Insects

Flying insects have the highest mass-specific aerobic metabolic rate among all animals (Snelling et al., 2012). Thermoregulation is used by foraging honeybees to optimize energetic costs under different environmental temperatures. When foraging in shade, they show a high level of energy turnover to speed up the foraging process, which is decreased by 18–76% under solar heat (Kovac et al., 2010; Stabentheiner and Kovac, 2014).

Studies have measured metabolic rate during maximal flight by the total amount of CO_2_ emitted (Mattila, 2015) or O_2_ consumption during a tethered flight (Snelling et al., 2012). Muscle efficiency increases with the increase in force production, whereas aerodynamic efficiency of lift decreases with the increase in force. Reduction in wing size of insects is associated with reduced flight muscle mass, mechanical power production, and overall flight efficiency. Therefore, insects are less efficient at converting metabolic power to mechanical power, and the total flight energetic efficiency of *Drosophila* is only 2–3% due to the high whole-body metabolic rate (Lehmann, 2001). The primary energy supply in insect muscles is from fat, and fat body cells control the utilization of energy from fat and glycogen (Arrese and Soulages, 2010).

Ca^2+^ cycling across plasma and SR membranes during the contraction and relaxation of vertebrate striated muscles and synchronous IFM of insects consumes large amounts of energy using ∼30% of the total ATP produced in muscle cells (Szentesi et al., 2001). In contrast, asynchronous IFM of insects works without beat to beat Ca^2+^ cycling, demonstrating a unique energy saving mode for generating high power work for flight (Conley and Lindstedt, 2002).

The cycling of Ca^2+^ during muscle contraction and relaxation by SR and plasma membrane Ca^2+^ pumps requires significant energy expenditure and consumes a large amount of ATP. Studies have estimated that mammalian cardiomyocytes use ∼30% of total ATP produced to drive the Ca^2+^ pumps (Starling et al., 1998). Studies of human skeletal muscles showed that the SR ATPase activity consumes equal amount of total ATP to that of myosin ATPase in slow twitch type I and approximately half of that in fast twitch type IIA fibers (Szentesi et al., 2001). By eliminating the need for high frequency cycling of large quantities of Ca^2+^ during each contraction and relaxation episode, the amounts of SR and membrane Ca^2+^ pumps are minimized in asynchronous IFM of insects, resulting in less ATP consumption versus that used by the myosin motors during powered flight (Conley and Lindstedt, 2002). Therefore, reducing the cost of Ca^2+^ handling in myocytes is a potential direction to increase the energetic efficiency of vertebrate striated muscles.

Myofilament regulatory proteins, especially Tn, play central roles in regulating the kinetics of force production, and therefore, contribute to the energetic efficiency of striated muscles. TnC is the Ca^2+^ receptor subunit of Tn. During muscle activation, the amount of intracellular Ca^2+^ directly determines the dynamic activation of TnC and the production of force. Insect muscles have two isoforms of TnC. The F1 form is ubiquitously expressed in all muscles and has only one Ca^2+^ binding site like that in vertebrate cardiac/slow TnC, whereas the F2 form is IFM-specific and has two Ca^2+^ binding sites like that in vertebrate fast TnC (Herranz et al., 2005). F2 TnC is important for stretch activation of IFM, and the higher proportion of F2 TnC, the higher the frequency that IFM can achieve (Herranz et al., 2005).

**FIGURE 4:**
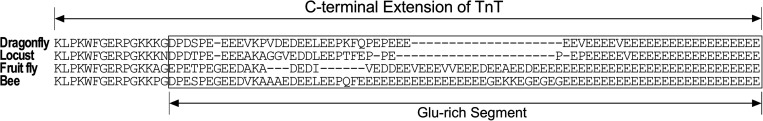
Glu-rich C-terminal extension of insect TnT. Amino acid sequences encoded by exon 11 or exon 12 of representative insect TnT genes are aligned to identify residue similarity and to illustrate the insect-specific C-terminal extension enriched with Glu residues. The Glu contents appear positively correlating to the frequencies of wing beat of these species (Snelling et al., 2012). GenBank accession numbers of the sequences used are: Dragonfly TnT, AAD33604.1; locust TnT, AVCP010016941; *Drosophila* TnT, NP_525088; Bee TnT, NP_001035348.1.

**FIGURE 5:**
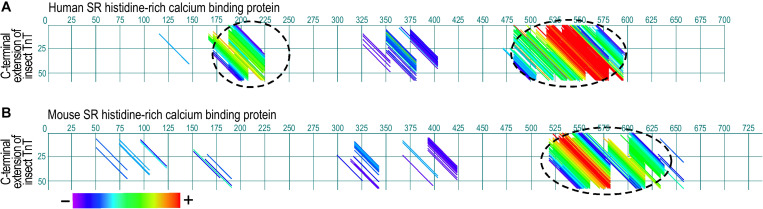
Paired dot plots of amino acid sequence similarities between the Glu-rich segment of insect TnT and sarcoplasmic reticulum histidine-rich calcium binding protein (HRC). Analyzed using DNAStar MegAlign Dotplot software method of pairwise alignment, the C-terminal extension of bee TnT matches with high similarity to segments in human **(A)** and mouse **(B)** HRC (indicated with dashed circles), suggesting an analogous function as a myofilament Ca^2+^ reservoir. The matched regions and degree of sequence similarity are indicated by the different colors of the diagonal lines. Shown in the color bar below the panels, red represents the highest similarity while purple shows the least similarity. The GenBank accession numbers for the sequences used are: Human HRC binding protein, AAH94691.1; mouse cardiac HRC protein, AAD42061.1; Bee TnT, PBC29029.1.

Insect muscle TnT has a long glutamic acid-rich C-terminal extension (Figure 4) that is not present in vertebrate TnT. Although it is not a part of the conserved core structure of TnT, deletion of the Glu-rich C-terminal extension of *Drosophila* TnT significantly decreased muscle and heart functions (Cao et al., 2020). As a potentially analogous structure, the *N*-terminal variable region of vertebrate TnT, especially the fast TnT in adult avian pectoral muscles with multiple P exons spliced-in (Ogut et al., 1999; Jin and Samanez, 2001), is also Glu-rich and binds Ca^2+^ with physiologically relevant affinity (Zhang et al., 2004), suggesting a function as a myofilament-associated Ca^2+^ buffer/reservoir. Supporting the analogous function, dot plot analysis of amino acid sequences revealed significant similarities between the Glu-rich C-terminal extension of bee TnT and the SR histidine-rich calcium binding protein (HRC) (Figure 5), which is a known Ca^2+^ buffer/reservoir in vertebrate muscle cells (Arvanitis et al., 2011). A hypothesis to be tested is that this Ca^2+^ buffer/reservoir provides a mechanism for local cycling of Ca^2+^ with TnC during the energy-saving stretch activation–deactivation cycles when TnC undergoes conformation-modulated changes in Ca^2+^-binding affinity.

### Birds

Measuring wingbeat kinematics as mechanical power and metabolic power (oxygen consumption) estimated that the efficiency of avian flight muscle is between 13% and 23% in small birds (Ward et al., 2001; Askew and Ellerby, 2007). Avian pectoral muscles power wing strokes with a significant fraction of shortening (33–42%) during flight (Biewener, 2011). The capacity of birds to fly or maintain a position in the air is affected by body size with declined fight performance as body size increases. An explanation is that the rate of power production does not keep up with increasing body size, thus mass-specific power production decreases, although it is partially compensated by increasing the number of higher-power production glycolytic fibers (Askew et al., 2001). A “heart to fly” hypothesis proposed that an efficient cardiovascular system with a large heart is required for oxygen delivery to the pectoral muscles for powered flight (Altimiras et al., 2017). Consistent with this hypothesis, hovering flight only evolved in hummingbirds with small body size, which produces the most energetically expensive muscle work with the highest mass-specific metabolic rates in vertebrates (Evangelista et al., 2010).

Unlike insects and mammals, flying birds exhibit a high daily energy turnover and have a longer lifespan compared to mammals of similar body mass. Because of chronically elevated blood glucose and insulin resistance, avian flight muscles predominantly use lipid oxidation to power locomotion, which yields more energy than carbohydrate metabolism. To prevent free radical production under the high metabolic rate, mitochondria of sparrow pectoralis muscle have a much higher capacity of oxidizing fatty acids than mitochondria of rat hindlimb muscles. In addition, avian flight muscle has a high electron transport chain/oxidase ratio, indicating a low redox potential for the production of reactive oxygen species (Kuzmiak et al., 2012).

Migrant birds can sustain long distance flight non-stop without food or water intake. The metabolic rate of migrating birds is adjusted in real-time by their migration needs. They primarily use lipids and a minimal amount of protein as fuel during flight (Jenni-Eiermann, 2017). During stopovers, the first response to lower the metabolic rate is an increase of plasma free fatty acid to stop further lipolysis. Showing a convergent evolution of muscle metabolism in adaptation to migrating flight, migratory bats use a similar strategy to adjust fuel types to that of migrating birds (Table 1) (McNab and O’Donnell, 2018), although bats also rely on torpor between flights to save energy (Knight, 2019).

**TABLE 1:** Contractile features, metabolic supply, other energy expenditures and energetic efficiency of insect, avian, and bat flight muscles.

**Flying animals**	**Contractility features**	**Metabolic supply**	**Other energy expenditures**	**Energetic efficiency**	**Specific traits related to Ca^2+^-Tn regulation**
Insects	Stretch activation of asynchronous IFM	Fat body cells	Low SR volume; high oxidative capacity	Reduced cost of ATP for Ca^2+^ cycling by stretch activation	C-terminal extension of TnT as a Ca^2+^ reservoir/buffer
Birds	Hovering of hummingbird powered by maximizing muscle function	Lipid oxidization	High oxidative capacity	Reduced force production in high frequency wing beating of hummingbird	*N*-terminal variable region of TnT as Ca^2+^ reservoir/buffer
Bats	3D structure wings supported maneuvering	Fuel by exogenous sugars	High SR volume		

During embryonic and postnatal development of vertebrate animals, myofilament proteins in cardiac and skeletal muscles undergo isoform and/or splice-form switches in response to changes in functional demand. Alternative splicing produces a postnatal switch of fast skeletal muscle TnT splice forms to splice-out fetal exons in the *N*-terminal variable region (Wang and Jin, 1997; Ogut and Jin, 1998). In addition to that in other vertebrate species and in avian leg muscles, avian pectoral muscle fast TnT has a unique concurrent post-hatch developmental switch to splice-in multiple avian-specific P exons encoding a long Glu-rich segment to significantly increase the length of the *N*-terminal variable region (Ogut and Jin, 1998). This avian adult pectoral muscle fast TnT-specific structure is not expressed in avian leg muscles (Ogut and Jin, 1998) and is absent in the fast TnT of the flightless bird emu (Cao et al., 2020). However, it shows a striking sequence similarity to the Glu-rich C-terminal extension of insect TnT, suggesting an analogous function from convergent evolutionary selections for flight (Cao et al., 2020). The hypothesis that the Ca^2+^-binding capacity of the *N*-terminal segment of avian pectoral muscle fast TnT may serve as a Ca^2+^ buffer/reservoir for local cycling of Ca^2+^ with TnC and reducing the work and energy cost of Ca^2+^ cycling pumps is supported by the finding that the *N*-terminal Glu-rich segment of avian pectoral muscle TnT binds Ca^2+^ at physiological affinity (∼14 μM) (Zhang et al., 2004).

### Bats

Similar to birds, bats have a high metabolic rate and relatively small body size but an exceptional longevity. Unlike birds, bats do not use triacylglycerol as oxidative fuel due to the lack of efficient transport enzymes in mammals (McGuire et al., 2013). Therefore, bats utilize ingested nutrients directly and rapidly, a possible reason for their inability to sustain long distance flights like birds. Metabolism of dietary sugar is the direct energy supply for nectarivorous bats to fly. In contrast, insects-feeding bats use protein-rich diet as a major energy source with rapid oxidation to fuel the high energy demand of flight (Table 1) (Voigt et al., 2017).

The high metabolic rate of bats is essential for their flying ability but also results in a systemic accumulation of harmful free radicals. Genomic studies found that bats evolved genetic variants to counter the toxicity of free radicals generated during flight (Jebb et al., 2018). It has been observed that the extremely high metabolic rate of bats during flight activities may impose a constant stress to stimulate the immune system. The genetic variants that minimize free radical damage might also contribute to bats’ unique immune homeostasis that allows them to be a natural reservoir of many viruses that are severely pathogenic in other mammals (Wang and Anderson, 2019).

A proposed mechanism of energetic efficiency in bats is the trade-off with maneuverability. Wind tunnel experiments showed that while bats are less energetically efficient than birds in cruising flight, they have the edge over birds when it comes to maneuvering (Table 1). While birds have reduced weight of wings due to the fusion of bones, bat wings have joints for 3D flapping to enhance aerodynamic force production (Hedenstrom and Johansson, 2015).

**FIGURE 6:**
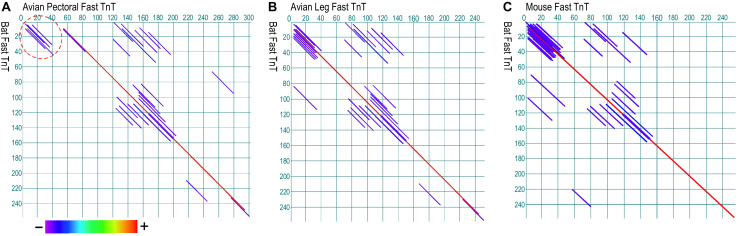
Paired dot plots of amino acid sequence similarities between TnT from bat fast twitch skeletal muscle, avian pectoral and leg muscles, and mouse fast twitch skeletal muscle. Analyzed using Dotplot method of DNAStar MegAlign software, bat TnT lacks the Glu-rich *N*-terminal segment found in avian pectoral TnT (**A**, indicated by the dash circle) whereas it has similar overall structure to that of avian leg muscle TnT **(B)** and mouse fast TnT **(C)**. Shown in the color bar below the panels, red represents the highest similarity while purple shows the lowest similarity. The GenBank accession numbers for the sequences used are: Bat fast skeletal muscle TnT, XP_028371248.1; avian pectoral fast skeletal TnT, AAG44258.1; avian leg muscle TnT, BAC76600.1; mouse fast skeletal muscle TnT, AAB67285.1.

To investigate whether the analogous Glu-rich segments evolved in insect and avian pectoral muscle TnT (Cao et al., 2020) had also convergently emerged in bat flight muscles as a potential mechanism to reduce the energy expenditure, dot plot analysis of bat fast TnT did not find significant structural similarity to the *N*-terminal Glu-rich region of avian pectoral muscle fast TnT, whereas its overall structure is conserved similar to that of avian leg muscle fast TnT and mouse fast skeletal muscle TnT (Figure 6).

## Biomedical Perspectives

By comparing the flight muscles of insects, birds, and bats for their evolution of contractile features and mechanisms for energetic efficiency, the data summarized in this focused review show that while these phylogenetically diverse animals independently evolved flight abilities at different times from different ancestral lineages, natural selection has yielded analogous flight functions. While many hypotheses and debated viewpoints remain for the origins of flight, the existing evidence provides informative insights into the understanding of muscle contractility and energetic efficiency. To achieve and sustain flight, all flying species need to balance flight performance and energy saving, which is also important in other muscles especially when the treatment of muscle weakness and heart failure is considered.

A better understanding of the molecular evolution of myofilament proteins in the context of physiological features of invertebrate and vertebrate flight muscles can help explore potential mechanisms and translational applications for improving human health. One intriguing point is the underlying molecular mechanisms for bird and bat flight muscles to function with very different metabolic rates reflecting different energetic efficiencies, although they have similar contractile machinery and under the selection of similar natural environments.

Another attractive direction for future research is that the low contractile efficiency of insect asynchronous IFM is sustained by minimizing the work and energy expenditure of the Ca^2+^ handling system that is a major ATP consumer in vertebrate striated muscles (Starling et al., 1998; Szentesi et al., 2001). Past and recent research on treating muscle weakness and heart failure has largely focused on increasing contractility and/or increasing ATP production in myocytes, which have limited potential and often decrease energetic efficiency restricting the long term benefit. Therefore, selectively reducing the energy expenditure of Ca^2+^ handling vs. that of myofilament myosin motors in mammalian striated muscle is an attractive direction to increase the energetic efficiency for the treatment of muscle weakness and heart failure.

## Author Contributions

Both authors contributed to the literature review, data analysis, compose figures and table, draft and revision of the manuscript.

## Conflict of Interest

The authors declare that the research was conducted in the absence of any commercial or financial relationships that could be construed as a potential conflict of interest.
